# Could cash and good parenting affect child cognitive development? A cross-sectional study in South Africa and Malawi

**DOI:** 10.1186/s12887-017-0883-z

**Published:** 2017-05-12

**Authors:** Lorraine Sherr, Ana Macedo, Mark Tomlinson, Sarah Skeen, Lucie Dale Cluver

**Affiliations:** 1 Research Department of Global Health, University College London, Rowland Hill Street, London, NW3 2PF UK; 2 Department of Psychology, Stellenbosch University, Stellenbosch, South Africa; 3 Department of Psychiatry and Mental Health, University of Cape Town, Cape Town, South Africa; 4 Department of Social Policy & Social Intervention, Centre for Evidence-Based Intervention, University of Oxford, Oxford, UK

**Keywords:** South Africa, Malawi, HIV/AIDS, Cash Grant, Parenting, Child development

## Abstract

**Background:**

Social protection interventions, including cash grants and care provision have been shown to effectively reduce some negative impacts of the HIV epidemic on adolescents and families. Less is known about the role of social protection on younger HIV affected populations. This study explored the impact of cash grants on children’s cognitive development. Additionally, we examined whether combined cash and care (operationalised as good parenting) was associated with improved cognitive outcomes.

**Methods:**

The sample included 854 children, aged 5 – 15, participating in community-based organisation (CBO) programmes for children affected by HIV in South Africa and Malawi. Data on child cognitive functioning were gathered by a combination of caregiver report and observer administered tests. Primary caregivers also reported on the economic situation of the family, cash receipt into the home, child and household HIV status. Parenting was measured on a 10 item scale with good parenting defined as a score of 8 or above.

**Results:**

About half of families received cash (55%, *n* = 473), only 6% (*n* = 51) reported good parenting above the cut-off point but no cash, 18% (*n* = 151) received combined cash support and reported good parenting, and 21% (*n* = 179) had neither. Findings show that cash receipt was associated with enhanced child cognitive outcomes in a number of domains including verbal working memory, general cognitive functioning, and learning. Furthermore, cash plus good parenting provided an additive effect. Child HIV status had a moderating effect on the association between cash or/plus good parenting and cognitive outcomes. The association between cash and good parenting and child cognitive outcomes remained significant among both HIV positive and negative children, but overall the HIV negative group benefited more.

**Conclusions:**

This study shows the importance of cash transfers and good parenting on cognitive development of young children living in HIV affected environments. Our data clearly indicate that combined provision (cash plus good parenting) have added value.

## Background

HIV can affect children directly when they themselves are HIV positive or indirectly when their parent/s are HIV infected. Most child HIV infection occurs at birth. In addition to those born and acquiring HIV, other children are born HIV negative to an HIV positive mother – thereby exposed to both the virus, the treatment and an environment where HIV is in the family [1–5]. In high prevalence countries, high HIV-burden within communities may also affect children. Negative effects can be direct from HIV related illnesses or insult on the neurological system; or indirect by the myriad of consequences of HIV infection in the family [6] and community Many of the documented effects of HIV also have the potential to affect optimum child development. These include parental illness or death; parental mental health diagnosis, parenting distraction due to illness, medication demands, clinic visits and challenges with coping and adjustment. HIV in the family may herald economic strain as unemployment is elevated and scarce family resources may be diverted to adult care needs. Time and quality of attention may affect younger children where alternative caregivers are brought in, sibling care may be needed, and school attendance may be disrupted. HIV is also associated with stigma and this may have a consequential negative effect on the family and the child [7].

This complex array of challenges necessitates complex interventions. Yet interventions at scale are wanting [8]. Of particular concern is cognitive development, as this may affect the child’s ability to reach their full developmental potential, limit their access to education and subsequently have long term implications for their life opportunities [9] Some areas of cognitive development are crucial for interpersonal behaviours and indeed are the very skills needed for HIV prevention. For example, difficulties with executive functioning may hamper their skills of negotiation and decision making for HIV safe behaviours. Cognitive challenges can set up a cascade of longer term problems. It is well established that children who perform less well in school are more likely to drop out, not reach secondary school or complete secondary school and may gravitate to higher risk behaviours including sexual risk, behavioural risk (such as bullying and violence) alcohol and drug use, and economic risk in later life [10].There is evidence of cognitive delay in a number of domains for HIV positive children – although the data does show that not all HIV positive children are affected [11]. Recent systematic reviews have documented the consistent concerns regarding cognitive outcomes and HIV exposure [12, 13]. In addition there is a growing evidence base that children who are negative but exposed to HIV in utero also experience delay [4] but the biological and/or social mechanisms of such effects are unclear.

It is also well documented that poverty can affect child development either directly, by means of such factors as malnutrition, or indirectly by way of reduced stimulation, opportunity or access to learning [14]. One of the current interventions under scrutiny relates to social protection, with a particular focus on cash transfers. Emerging literature shows the efficacy of cash transfers on positive child outcomes [15, 16]. Some cash transfer studies have been conditioned on parental behaviours that may enhance child wellbeing, such as birth registration, immunisation, parenting class attendance and school enrolment [17, 18]. Unconditional cash transfers have also shown similar gains for children and these obviate the problems of dealing with those who fail to meet the conditions (perhaps the most in need) [19]. Some countries (such as South Africa and Lesotho) have managed to integrate cash transfers at a national level and the rollout of transfers has been incorporated into government planning [20].

A recent set of studies have examined specifically how cash transfers may reduce HIV risk behaviours and what additional inputs could enhance the efficacy of cash transfers [21, 22].In a study of adolescents, cash transfer receipt reduced a series of HIV-risk behaviours in girls (though not in boys) [23]. A further examination of this data showed that cash complemented with care was associated with halved HIV-risk behaviour for both girls and boys. . ‘Cash plus care’ has also been shown to reduce school dropout, violence perpetration and substance use amongst adolescents [22]. Care has been operationalised in studies of older children, and comprises elements such as absence of harsh punishment, good parenting, and school/community provision such as groups and psychosocial support.

Given that cash – and cash plus care – can affect adolescent risk behaviour, it raises the question of whether cash transfers given to families have anything to offer in terms of younger child cognitive development? Furthermore, could supplementing cash with good care provide additive protection, and if so, for which children? Very little information is available for younger children. Given their age they are less likely to access broader care avenues, but are highly reliant on good parenting within the home. This study aimed to explore: 1) potential effects of cash grants into the home on cognitive function in younger children; and 2) whether cash plus care (operationalised as good parenting) had any additive effects. A detailed analysis of different forms of cognitive performance and an exploration of a variety of vulnerability factors may provide insight into the role of cash transfers and quality of parenting for child development in high HIV affected environments in resource poor settings.

## Methods

### Participants

The sample included children between the ages of 5 and 15 years and their primary caregivers. Data were collected between 2013 and 2014 as part of the Child Community Care project, a study tracking the development of children and families affected by HIV attending established community based organisations (CBOs) across South Africa and Malawi. Eleven partner organisations (AIDS Alliance, Stop AIDS Now, Diana Memorial Fund, Firelight Foundation, Bernard van Leer foundation, REPSSI, World Vision, Comic Relief, Help Age, Save the Children and UNICEF) provided a list of all their funded CBOs. The list comprised 588 CBOs (524 in South Africa and 64 in Malawi). All 588 CBOs were stratified by funding partner and geographical location and 28 (24 in South Africa and 4 in Malawi) were randomly selected. All 28 CBOs agreed to participate in the study. Ethical approval was obtained from the ethics boards of University College London Research Ethics Committee (reference number 1478/002) and Stellenbosch University Health Research Ethics Committee (reference number N10/04/112) and authorised by each of the funding partners of the various community-based programmes. Caregivers received full information on the study and gave written consent for their own and their child’s participationon a specially developed informed consent form translated into local languages. Children were given information about the study in child-friendly local language and provided written assent on an assent form by writing their names or making another mark.

### Procedure

Data on the children were gathered by a combination of self-report and caregiver report. Questionnaires (for the child and caregiver) included a range of questions and standardised measures related to child’s health, education, psychosocial wellbeing, cognitive functioning and socio-demographic information. Questionnaires were translated into Zulu and Xhosa and converted to mobile phone technology for ease of data collection and to allow for live monitoring [24]. Children and caregivers were interviewed separately by trained data collectors and all data were entered live into mobile phones and captured via the Mobenzi system into a database. The cash transfer questions were available at time 2 of the data collection exercise (2013-2014) and were utilised in this analysis. At recruitment refusal rates were low (.7%).

### Measures

#### Demographic and socio-economic characteristics

Children’s age, gender, HIV status and access to HIV treatment were determined by caregiver report. Number of household assets was used as an indicator of household wealth and was drawn from the Demographic and Health Survey (DHS) household questionnaire [25]. Caregivers were asked to indicate how many of the following 10 items they owned: refrigerator, stove, television, radio, telephone, mobile phone, computer, internet, car, and bicycle. The household asset scale ranged between 0 and 10 with higher scores indicating greater number of assets. Caregivers were also asked to indicate which of the different types of houses they lived in (i.e., house/flat, a shack, on the street), and responses were dichotomised into informal versus formal housing.

### Cash grant receipt

Caregivers reported on whether they received one or more of the following six grants into the home: a retirement pension, state pension, disability grant, child support grant, foster care grant, or care dependency grant. Grant receipt was dichotomised into those receiving any grant versus none. **Number of grants** available to families ranged from 0 to 6, with some grants being mutually exclusive depending on household situation.

### Parenting

Good parenting was operationalised based on a composite index of 10 items with a binary yes/no score. Children were asked four questions - whether they felt they belonged with the people at home, received praise, received treats and whether adults hugged as well as praised them (drawn from items of the Child Status Index tool [26]). Caregivers reported on 6 items – the use of positive discipline styles (explaining to the child when they did wrong deeds, taking away privileges as opposed to harsh punishments, and beatings), provision of consistent care, and absence of physical or emotional violence towards the child (drawn from items of the Parent-Child Conflict Tactics Scale [27]). A scale ranging from 0 to 10 was generated with 0 being the lowest score and 10 the highest score. The good parenting measure was then dichotomised to those scoring above 8 (*n* = 101) reflecting “good-enough parenting” and those scoring 7 or below (*n* = 732). This cut-off was chosen to reflect a high enough standard of parenting, as no participants scored 10, and only 1 caregiver scored 9 [28].

### Outcomes

Five cognitive measures were employed in this study. Two were based on standardised tests which were administered by a fully trained objective data collector. Three were based on caregiver report according to a standardised disability inventory. These included the **Draw-a-person (DAP) Test**, a screening test used as an indicator of nonverbal cognitive ability based on children’s drawings of human figures [29]. Children were asked to draw a picture of themselves, a man, and a women. Drawings were then assessed using the Draw-a-Person Quantitative Scoring System (QSS), which analyses 14 different aspects of the drawings, such as specific body parts and clothing, for various criteria, including presence or absence, detail, and proportion. Overall, there are 64 scoring items for each drawing. All drawings were coded and marked by a researcher who was blinded to the child’s identity at the time of assessing the drawings. An age-standardised score was recorded for each drawing, and mean scores were calculated (scale ranges 40-130). There are few cognitive screening tools for young children in Sub-Saharan Africa and this test was considered the most appropriate. This revised version of DAP has been previously used in African countries [30–32]. Additionally, the use of a nonverbal, quick and easy-to-administer task has the advantage of eliminating potential sources of bias, including primary language, verbal skills, or communication difficulties. The **Digit Span Test** is a subtest of the Wechsler Intelligence Scale for Children (WISC-IV) and measures attention and working memory [33]. The test consists of repeating dictated series of digits (e.g., 4 1 7 9) forwards and other series backwards. Series begin with two digits and keep increasing in length with two trials at each length. A total scaled score for the two recall conditions was computed (range 0-20). The scaled score is an age-based, norm referenced score for each child, based on a large nationally representative norm sample of South African children [34]. Primary caregivers were asked to report on **child functioning and disability** in three cognitive domains: **learning, remembering new things, and comprehension**. These questions were taken from a newly developed disability measure [35] for use in low and middle income settings. Ratings were in a *3-*point difficulty scale*: 0 (no difficulty), 1 (some difficulty), 2 (a lot of difficulty), 3 (cannot do at all).* Mean scores were computed for each domain, and a total score was calculated for all 3 domains combined.

### Statistical analysis

A five-stage analysis strategy was carried out in IBM SPSS 22.0. First, we looked at differences between those receiving a cash grant (at least one of six possible grants into the family) and those who received no grant at all on demographic variables and five cognitive measures: non-verbal cognitive ability (assessed using draw-a-person test), short-term memory/attention (measured using digit span test), and difficulty or disability in three cognitive domains: learning, remembering new things, and comprehension. Second, we examined associations between quality of parenting and child cognitive outcomes. Third, a cumulative “cash and good parenting” scale was hypothesised: no support (0), cash grant receipt(1), good parenting (based on existing evidence of impacts of positive parenting) (2), integrated cash and good parenting (3), and coded both as ordinal and as dummy variables for use in regression models. A series of ANOVA analyses tested associations between types of provision (cash, good parenting or both) and all five cognitive measures. Fourth, a series of linear regression models were used to further examine associations of cash, good parenting, and combined provision (represented by dummy variables, taking “none” as the reference category) with cognitive outcomes. Model 1 shows unadjusted associations between types of social protection and cognitive outcomes and Model 2 included potential co-factors predicting either cognitive development or receipt of social protection (child gender, age, HIV status functioning or disability, and number of household assets). Draw-a-person and digit span tests are age-adjusted, thus child age was not included as a covariate in multivariate regression analyses. Fifth, regression analyses disaggregated by HIV status and using interaction terms were used to examine whether receiving cash support, having good parenting or both had differential effects on cognitive outcomes of HIV positive and HIV negative children.

## Results

### Socio-demographic characteristics and child cognitive development by cash grant receipt

Data from a total of 854 children in South Africa (*n* = 708) and Malawi (*n* = 146) were analysed. 52.3% were female, and ages ranged from 5 to 15 years (M = 10.19, SD = 2.81). Primary caregivers reported that 13.5% of children (*n* = 115) were HIV positive. Of those, 112 (97.4%) were receiving medical treatment. Overall, 108 children (13.3%) were living in informal dwellings and most households lacked essentials such as a refrigerator or a stove (mean of 3.90 out of 10 household assets). Of the six possible grants available to families, 60.9% of caregivers reported they received just one grant (*n* = 520), 7.4% received two, and only 0.2% received three. 73.1% of caregivers (*n* = 624) reported receiving at least one cash grant; yet, 26.9% reported no cash grant at all, despite the fact that socio-economic status indicators showed high levels of deprivation.

Grant receipt according to HIV status of the child showed that HIV positive children were less likely to get a cash grant compared to HIV negative children (60.0% versus 75.3%, X^2^(1) = 11.89, *p* = 0.01). Differences between children residing in households receiving a grant and those not receiving are set out in Table 1 below.

**Table 1:** Sample characteristics by cash grant receipt (any grant vs. no grant into the child’s household)

	Total (*n* = 854)	Grant (*n* = 624)	No grant (*n* = 230)	X^2^ or F (df), *p* value
Country				
South Africa	708 (82.9%)	624 (88.1%)	84 (11.9%)	477.8 (1), *p* < 0.001
Malawi	146 (17.1%)	0	146 (100%)	
Child gender				
Boy	400 (47.7%)	289 (72.3%)	111 (27.8%)	0.13 (1), *p* = 0.76
Girl	439 (52.3%)	322 (73.3%)	117 (26.7%)	
Child age	10.21 (2.81)	9.99 (2.80)	10.80 (2.73)	14.02 (1), *p* < 0.001
Child HIV status				
HIV positive	115 (13.5%)	69 (60.0%)	46 (40.0%)	11.89 (1), *p* = 0.01
HIV negative or unknown	737 (86.5%)	555 (75.3%)	182 (24.7%)	
Home				
Living in a house or flat	689 (86.6%)	481 (69.8%)	208 (30.2%)	13.47 (1), *p* < 0.001
Living in a shack	107 (13.4%)	93 (86.9%)	14 (13.1%)	
N of household assets	3.90 (1.93)	2.60 (2.16)	4.38 (1.58)	173.15 (1), *p* < 0.001
Child cognitive outcomes				
Draw-a-person test	91.25 (17.28)	95.29 (14.92)	80.34 (18.47)	144.90 (1), *p* < 0.001
Digit span test	8.97 (3.56)	9.34 (3.54)	7.98 (3.44)	24.28 (1), *p* < 0.001
Learning difficulty	0.20 (0.47)	0.15 (0.43)	0.33 (0.56)	26.43 (1), *p* < 0.001
Remembering difficulty	0.34 (0.58)	0.31 (0.56)	0.42 (0.63)	6.68 (1), *p* = 0.01
Comprehension difficulty	0.04 (0.24)	0.04 (0.20)	0.07 (0.32)	3.91 (1), *p* = 0.048
Total cognitive difficulties	0.58 (1.04)	0.49 (0.94)	0.83 (1.24)	17.99 (1), *p* < 0.001

Cognitive outcomes were measured for all children using the digit span test, the draw a person test and three items from the UNICEF disability inventory (learning, remembering new things and comprehension). The mean score for the Draw-a-Person test was 91.25 (SD = 17.28) which falls within the norm group scores (ranging between 90 and 109). A total of 361 children (43.3%) had scores below the normative scaled score mean of 90. The mean Digit Span scaled scores for the entire group was 8.97 (SD = 3.56). Less than half of children (44.8%, *n* = 371) had scores at or below the normative scaled score mean of 10 [33]. Children scored low in the severity scale for the three cognitive disability domains: mean for learning difficulty was 0.20 (SD = 0.47), mean for remembering new things difficulty was 0.34 (SD = 0.58), and mean for comprehension difficulty was 0.04 (SD = 0.24). Children in households receiving grants showed better cognitive outcomes as set out in Table 1 below.

### Associations between good parenting and child cognitive outcomes

A total score on 10 dimensions of parenting provided for a working definition of good parenting with 0 being the lowest score and 10 the highest score. The mean score of the parenting scale was 6.46 (SD = 0.98), and higher scores were significantly associated with better cognitive outcomes. More specifically, higher parenting scores were associated with better performance on draw-a-person test (B = 1.98, 95% CI: .79, 3.17, *p* = .001), and on digit span test (B = .37, 95% CI: .13, .62, *p* = .003). Higher scores on the parenting scale were also positively associated with less severity in learning difficulty (B = −.049, 95% CI: −.08, −.02, *p* = .003), and less severity in remembering difficulty (B = −.06, 95% CI: −.10, −.20, *p* = .003). There was no difference according to parenting score on comprehension difficulty score. For the purpose of the next set of analyses, good parenting was dichotomised to those scoring above 8 (*n* = 101) seen as good parenting group, and those scoring 7 or below (*n* = 732) as not good parenting, and consequently a cut-off of 8/10 was chosen to reflect ‘adequate parenting’ as no caregivers scored 10/10 and only 1 caregiver scored 9/10.

### Associations between cash grant receipt plus having good parenting with children’s cognitive development

**Fig. 1:**
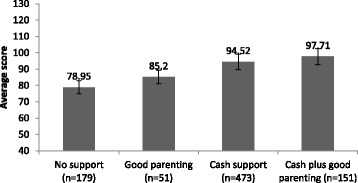
Associations between social protection access and cognitive performance on Draw-a-person test, F(3) = 52.31, *p* < .001

**Fig. 2:**
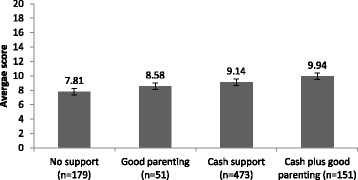
Associations between social protection access and performance on digit span test, F(3) = 10.67, *p* < .001

**Fig. 3:**
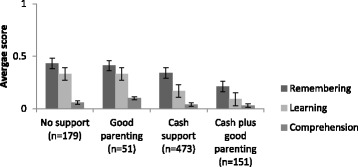
Associations between social protection access and difficulties in remembering (F(3) = 3.99, *p* = .008), learning (F(3) = 9.92), *p* < .001), and comprehension (F(3) = 1.68, *p* > .05)

Of the total sample, more than half of children lived in households receiving cash support (55.4%, *n* = 473), only 6% of children (*n* = 51) received care above the cut off point for good parenting but no cash, 17.7% (*n* = 151) received combined cash support and had good parenting, and 179 (20.9%) received none of those. A series of univariate ANOVA analyses tested associations between types of social protection and five cognitive measures: non-verbal cognitive ability (assessed using draw-a-person test), short-term memory/attention (measured using digit span test), and difficulty or disability in three cognitive domains: learning, remembering new things, and comprehension. For all cognitive outcomes, apart from the comprehension difficulty score, cash plus parenting above the cut-off was associated with better outcomes. Statistically significant associations are illustrated in Figs. 1, 2 and 3. As shown in Figs. 1 and 2, as provision increased from no support to cash plus good parenting, child cognitive performance improved. Cash plus good parenting access was also positively associated with less severity in two cognitive difficulty/disability domains: learning and remembering new things (see Fig. 3).

Unadjusted linear regressions examined associations of cash, care, and combined cash plus good parenting (Table 2) (represented by dummy variables, taking “no support” as the reference category) with all cognitive outcomes measured (Model 1). Compared with no support, cash receipt was associated with better performance on draw-a-person test (scaled scores ranged between 40 and 130) (B: 15.57; 95% CI 12.81-18.33, *p* < .001) and cash plus good parenting was associated with greater performance (B: 18.66; 95% CI 15.17 - 22.15, *p* < .001). Cash receipt was also associated with higher scores on digit span test (scaled scores ranged from 0 to 20) (B: 1.33; 95% CI: .72-1.95, *p* < .001), and cash plus good parenting was associated with an almost twofold improved score (B: 2.13; 95% CI 1.35-2.90, *p* < .001). Compared to no support, receiving cash was associated with lower scores in learning difficulty (B: −.17; 95% CI: −.25, −.09, *p* < .001), and cash plus good parenting was associated with the lowest level of difficulty (B: −.24: 95% CI: −.34, −.14, *p* < .001). Receiving cash plus good parenting was also associated with lower scores in remembering difficulty (B: −.21; 95% CI: −.34, −.09, *p* = .001). When combining the three indicators into an overall score of cognitive difficulty, we found that receiving cash was associated with lower difficulty scores (B = −.27, 95% CI: −.45, .09, *p* = .003), and that cash plus good parenting was associated with a greater reduction in cognitive difficulties (B = −.47, 95% CI: −.70, 95% CI: −.70, .25), *p* < .001).

**Table 2:** Linear regression models showing predictors of children’s cognitive outcomes

	Performance on cognitive tests		Cognitive functioning difficulty or disability			
	Draw-a-person	Digit span	Learning	Remembering	Comprehension	Total difficulties
	*B (95% CI)*	*B (95% CI)*	*B (95% CI)*	*B (95% CI)*	*B (95% CI)*	*B (95% CI)*
Model 1						
Cash support	15.57 (12.81, 18.33)***	1.33 (.72, 1.95)***	−.17 (−.25, −.09)***	−.009 (−.19, .01)	−.03 (−.07, .02)	−.27 (−.45, −.09)**
Good parenting	6.25 (1.25, 11.24)*	.77 (−.33, 1.87)	.004 (−.14, .15)	−.01 (−.19, .17)	.04 (−.04, .11)	.03 (−.29, .35)
Cash plus good parenting	18.66 (15.17, 22.15)***	2.13 (1.35, 2.90)***	−.24 (−.34, −.14)***	−.21 (−.34, −.09)***	−.04 (−.09, .02)	−.47 (−.70, −.25)***
Model 2						
Cash support	12.37 (9.42, 15.33)***	.85 (.20-1.51)*	−.09 (−.18, .001)	−.02 (−.13, .09)	.02 (−.03, .06)	−.09 (−.28, .10)
Good parenting	6.18 (1.31, 11.04)**	.79 (−.28, 1.87)	.008 (−-.14, .15)	−.01 (−.19, .17)	.05 (−.03, .12)	.04 (−.27, .35)
Cash plus good parenting	16.01 (12.45, 19.57)***	1.73 (.94, 2.51)**	−.17 (−.28, −.06)**	−.13 (−.27, .001)*	.003 (−.05, .06)	−.30 (−.53, −.07)**
Child gender (female)	.48 (−1.64, 2.60)	.19 (−.28, .66)	−.09 (−.15, −.02)**	−.09 (−.17, −.01)	−.03 (−.06, −.001)	−.21 (−.35, −.07)*
Child age (years)	-	-	.001 (−.01, .01)	.009 (−.005, .02)	−.03 (−.06, −.001)*	.008 (−.02, .03)
Number household assets	1.35 (.74, 1.96)***		−.004 (−.05, .02)***	−.03 (−.05, −.007)*	−.01 (−.02, −.004)**	−.08 (−.12, −.04)***
Child HIV status (HIV+)	-6.49 (−9.64, −3.35)***	−.92 (−1.61, −.22)*	.11 (.01, .20)	.16 (.04, .28)**	.08 (.04, .13)***	.35 (.15, .55)**
Child functioning difficulty or disability	−.64 (−1.09, −.19)**	−.25 (−.35, −.15)***	-	-	-	-
Interactions						
HIV x Cash	−3.87 (−8.48, .75)	−1.20 (−2.22, −.17)*	.02 (−1.12, .15)	.13 (−.04, .30)	.05 (−.02, .11)	.19 (−.10, .49)
HIV x Good parenting	−11.01 (−21.29, −.73)*	−1.91 (−4.25, .44)	.22 (−.08, .52)	.33 (−.04, .71)	.20 (.05, .35)**	.75 (.09, 1.41)*
HIV x Cash plus good parenting	.52 (−7.98, 9.00)	−1.27 (−3.14, .61)	−.03 (−.27, .22)	.05 (−.26, .36)	.04 (−.08, .17)	.07 (−.48, .61)

B: unstandardised coefficient, CI: confidence interval

Model 1: Univariate regression analyses showing associations of cash, good parenting and combined cash and good parenting with cognitive outcomes; Model 2: Multivariate regression analyses showing associations of cash, good parenting and combined cash and good parenting with cognitive outcomes controlling for other predictors: child gender, age, HIV status, number of household assets, and functioning difficulty or disability

*p* < .05, *p < .01, ** *p*< .001 ***

Interactions: *p* value refers to interaction of child HIV status and 3 types of provision: cash support, good parenting, and cash plus good parenting

In multivariate linear regressions (Model 2, Table 2), after controlling for factors predicting cognitive development or receipt of cash plus having good parenting (child gender, age, HIV status, functioning or disability, and number of household assets), combined cash plus good parenting remained a strong predictor. Children receiving cash plus having good parenting had higher scores, both on draw a person test (B: 16.01; 95% CI12.45-19.57, *p* < .001) and digit span test (B:1.73; 95% CI.94, 2.51, *p* < .001). Being HIV positive and having a disability also remained significant predictors of cognitive performance. After adjusting for significant cofactors, receipt of cash was no longer associated with cognitive difficulties, but combined cash and good parenting was significantly associated with lower scores of cognitive difficulties (B: −.30, 95% CI: −.53, −.07, *p* < .001), and in particular with lower severity scores in learning difficulty (B: −.17; 95% CI: −.28, −.06, *p* = .02) and difficulty in remembering new things (B: −.13, 95% CI: −.27, −.001, *p* = .04). No significant effect for comprehension was found.

### Moderating effect of HIV status on the association of cash and parenting with child cognitive function

HIV positive children had a significantly poorer performance in cognitive tests and greater difficulty/disability scores compared to the HIV negative group. In a series of linear regressions using interaction effects, we tested whether the effects of cash or/ and good parenting on cognitive outcomes differed by child HIV status (Table 2). For draw-a-person test and compared to no support, receiving cash was associated with better performance in both groups. Good parenting had a positive impact on performance for the draw-a-person test, particularly amongst HIV positive children (B = 9.83, (95% CI: -1.25, 20.92) compared to HIV negative children (B = 5.89, 95% CI: 5.89, 95% CI: .35, 11.43)*p* = 0.036. Cash plus good parenting had an additive effect on cognitive performance in both groups. Receiving cash was also associated with better performance in the digit span test, in particular for the HIV negative group (B = 1.34, 95% CI: 1.34, 95% CI: .67, 2.01) compared to HIV positive children (B = .90, 95% CI: -2.63, 2.46), *p* = .02. For the cognitive components in the disability measure (learning, remembering and comprehension difficulty), as provision increased from no support to cash plus good parenting, difficulty severity scores were reduced for both groups. We also noted that good parenting was associated with lower comprehension difficulty for the HIV negative children (B = .02, CI: −.04, .09) compared to the other group (B = .10, 95% CI: −.18, .38), *p* = .008, and also a lower overall cognitive difficulty score, particularly amongst the HIV negative group (*p* = .03).

### Effects on the most vulnerable children

Vulnerable children (Table 3) were defined as being HIV infected, boys and girls living in informal housing, and those with a disability. For receipt of cash alone, there were no differences by gender and disability, but higher likelihood of cash receipt amongst children in South Africa (66.8%, *p* < .001), informal dwellers (69.2%, *p* = .001) and younger children (aged 5 to 9) (59.8%, *p* = .04). HIV positive children were significantly less likely to live in households receiving a cash grant (45.2%, *p* = .02); yet they were more likely to receive better care (good parental practices) (10.2%, *p* = .03). Overall, only 151 children (17.7%) received combined cash support and good care. Children with a disability were more likely to receive cash plus care (19.4%), but there were no differences amongst other risk groups (HIV infected, informal dwellers, or younger age).

**Table 3:** Number and proportion of children receiving types of social protection by country, gender and high-risk group

	South Africa (*n* = 708)	Malawi (*n* = 146)	p	Girls (*n* = 439	Boys (*n* = 400)	p	HIV+ (*n* = 115)	HIV- (*n* = 737)	p	Any disability (*n* = 547)	No disability (*n* = 307)	p	5-9 yrs. (*n* = 331)	10-15 yrs. (*n* = 500)	p	Informal housing (*n* = 107)	Formal housing (*n* = 689)	p
No support (*n* = 179)	63 (8.9%)	116 (79.5%)	<.001	91 (20.7%)	86 (21.5%)	n.s.	34 (29.6%)	143 (19.4%)	.02	106 (19.4%)	73 (23.8%)	n.s.	50 (15.1%)	124 (24.8%)	.001	9 (8.4%)	162 (23.5%)	<.001
Cash (*n* = 473)	473 (66.8%)	0	<.001	238 (54.2%)	337 (56.8%)	n.s.	52 (45.2%)	421 (57.1%)	.02	304 (55.6%)	169 (55.0%)	n.s.	198 (59.8%)	262 (52.4%)	.04	74 (69.2%)	358 (52.0%)	.001
Good parenting (*n* = 51)	21 (3.0%)	30 (20.5%)	<.001	26 (5.9%)	25 (6.3%)	n.s.	12 (10.4%)	39 (5.3%)	.03	31 (5.7%)	20 (6.5%)	n.s.	18 (5.4%)	33 (6.6%)	n.s.	5 (4.7%)	46 (6.7%)	n.s.
Cash plus good parenting (*n* = 151)	151 (21.3%)	0	<.001	84 (19.1%)	62 (15.5%)	n.s.	17 (14.8%)	134 (18.2%)	n.s.	106 (19.4%)	45 (14.7%)	.05	65 (19.6%)	81 (9.7%)	n.s.	19 (17.8%)	123 (17.9%)	n.s.

## Discussion

Our findings show notable levels of cognitive delay in this community sample – both in observer administered standardised cognitive tests and caregiver ratings. Cash grants are being rolled out, but at this time point despite availability, access was not universal especially amongst the most needy groups who were significantly less likely to receive the cash supplements they were entitled to. Ideally support in access is needed to ensure inclusion even when government rollout is in place. Our findings show that those with an HIV positive child were significantly less likely to get cash and this form of social protection may need to be linked to clinical care to enhance receipt.

Cash plus care has been established as an effective intervention for lowered adolescent HIV risk behaviour, and our data now extends this by providing evidence in an HIV affected environment showing the specific advantages of cash in the context of good parenting on cognitive functioning. The data clearly indicates that cash transfers are associated with improved cognitive outcomes. Furthermore cash plus good parenting enhances the effects. This holds true for memory (measured by digit span), overall cognition (measured by the draw-a-person test) and learning and recall as measured by caregiver report. Cash transfers are now available in both South Africa and Malawi. It was of note that accessing such transfers in Malawi was exceedingly poor despite the high level of need. Access in South Africa was higher, but those with well-established needs, such as HIV infected children, were still not in receipt of such grants. This and other evidence suggest the importance of ensuring that even the most vulnerable children receive cash transfer programmes.

Given the clear cumulative effect of cash plus good parenting, our data supports the roll out of cash transfers but suggests that enhanced social protection may be useful in extending the benefit. We also note that the particularly needy groups such as HIV infected, disabled or those in extreme poverty, can benefit specifically from cash and cash plus good parenting. Good parenting is a key ingredient of ensuring optimal child development. Parenting skills have been shown to be amenable to intervention and it is clear from our data that parenting interventions could be of benefit in these vulnerable community settings. In terms of cognitive delay, there are few scaled interventions that can improve cognitive performance. From the remedial educational literature there are a number of interventions, yet few are being translated and provided to these young children. Those that are established, such as cognitive rehearsal [36] operate at the individual level and may be quite costly to roll out at scale. Yet it is well established that there are cognitive effects of HIV on children and that provision of cash in the context of good parenting may be an additional and alternative possibility to be considered for scaled interventions.

The study is not without its limitations. Our study was a field study and as such a number of factors could not be controlled for. Despite a large sample, the subgroups may have been small and thus underpowered. The study was not a randomized controlled study and there may have been systematic bias in the field in terms of receipt of both cash and parenting. Future studies may need to test out these concepts in a more controlled trial to establish causal links. We confined our care measure to examine good parenting, but there are a number of additional care concepts that could enhance cash transfers and need to be tested in terms of their benefit. Our good parenting measure was generated by a combination of child and caregiver self-report and could have been more robust if a validated measure was used (yet these are predominantly self-report) or an observer rating was included. HIV status was based on caregiver report and not confirmed with laboratory testing. Such measures have been used reliably in the field, but underreporting may be a possibility and future research may include laboratory tests. There are limited validated tools available for screening for child development outcomes in Sub-Saharan Africa. The cognitive screening tools used in this study were validated for South African children only. No measure of amount was taken in terms of the cash grant and future studies may need to examine the size of the cash grant into the household. All six available grants were recorded, but some are mutually exclusive in practice and no additive impact was possible to examine in this study. Future work could compare different forms of grant to examine efficacy.

## Conclusion

In conclusion this data has specific implications for planning of provision and services for children infected and affected by HIV. Our findings show that the most vulnerable children are linked with lower cash and care receipt. It is unclear whether it is the vulnerability that is linked to non-receipt of cash, or that the non-receipt creates or compounds the vulnerability. The most likely explanation is perhaps both – that they act in a synergistic manner. Our data shows clear benefits of both cash and good parenting on cognitive measures for younger children – even in the presence of cognitive delay or disability. What our data do suggest is that fragile groups may need multiple support avenues. Our findings suggest that there is a is a clear role for parenting programs to be made available in conjunction with cash transfers to enhance the effects and stack the odds for cognitive development outcomes for young children in high HIV affected areas. This study was carried out in the context of HIV. Future studies are needed to evaluate the impact of cash and parenting programmes on other infectious and chronic diseases.

## Abbreviations

ANOVA: Analysis of variance
CBO: Community-based organisation
DAP: Draw-a-person test
DHS: Demographic and Health Survey
HIV: Human immunodeficiency virus
QSS: Draw-a-Person Quantitative Scoring System
SPSS: Statistical package for the social sciences
UNICEF: United Nations Children’s Fund
WISC-IV: Wechsler Intelligence Scale for Children
